# Oxidized Product Profiles of AA9 Lytic Polysaccharide Monooxygenases Depend on the Type of Cellulose

**DOI:** 10.1021/acssuschemeng.1c04100

**Published:** 2021-10-13

**Authors:** Peicheng Sun, Susana V. Valenzuela, Pimvisuth Chunkrua, Francisco I. Javier Pastor, Christophe V. F. P. Laurent, Roland Ludwig, Willem J. H. van Berkel, Mirjam A. Kabel

**Affiliations:** † Laboratory of Food Chemistry, Wageningen University & Research, Bornse Weilanden 9, 6708 WG Wageningen, The Netherlands; ‡ Department of Genetics, Microbiology and Statistics, Faculty of Biology, University of Barcelona, Av. Diagonal 643, 08028 Barcelona, Spain; § Institute of Nanoscience and Nanotechnology (IN2UB), University of Barcelona, Av. Diagonal 645, 08028 Barcelona, Spain; ∥ Biocatalysis and Biosensing Laboratory, Department of Food Science and Technology, BOKU−University of Natural Resources and Life Sciences, Muthgasse 18, 1190 Vienna, Austria; ⊥ Institute of Molecular Modeling and Simulation, Department of Material Sciences and Process Engineering, BOKU−University of Natural Resources and Life Sciences, Muthgasse 18, 1190 Vienna, Austria

**Keywords:** Biomass, Biorefinery, Cellulose, Auxiliary Activity (AA), Lytic polysaccharide monooxygenase (LPMO), Carbohydrate-binding module (CBM), Oxidized cello-oligosaccharide, Product profile

## Abstract

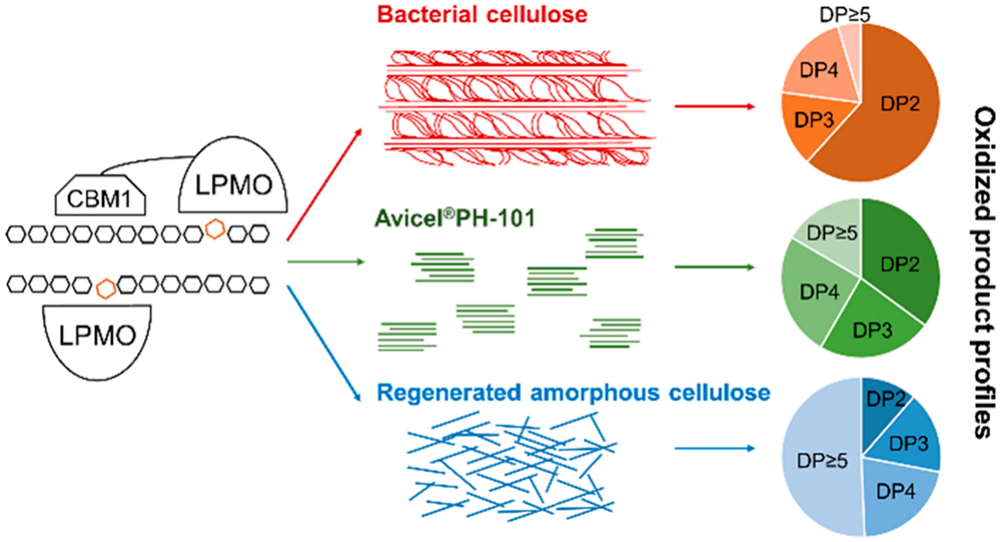

Lytic polysaccharide monooxygenases (LPMOs) are essential for enzymatic conversion of lignocellulose-rich biomass in the context of biofuels and platform chemicals production. Considerable insight into the mode of action of LPMOs has been obtained, but research on the cellulose specificity of these enzymes is still limited. Hence, we studied the product profiles of four fungal Auxiliary Activity family 9 (AA9) LPMOs during their oxidative cleavage of three types of cellulose: bacterial cellulose (BC), Avicel PH-101 (AVI), and regenerated amorphous cellulose (RAC). We observed that attachment of a carbohydrate-binding module 1 (CBM1) did not change the substrate specificity of LPMO9B from *Myceliophthora thermophila* C1 (*Mt*LPMO9B) but stimulated the degradation of all three types of cellulose. A detailed quantification of oxidized ends in both soluble and insoluble fractions, as well as characterization of oxidized cello-oligosaccharide patterns, suggested that *Mt*LPMO9B generates mainly oxidized cellobiose from BC, while producing oxidized cello-oligosaccharides from AVI and RAC ranged more randomly from DP2–8. Comparable product profiles, resulting from BC, AVI, and RAC oxidation, were found for three other AA9 LPMOs. These distinct cleavage profiles highlight cellulose specificity rather than an LPMO-dependent mechanism and may further reflect that the product profiles of AA9 LPMOs are modulated by different cellulose types.

## Abstract 2

AA9 LPMOs generate mainly oxidized cellobiose from bacterial cellulose but more evenly distributed oxidized cello-oligosaccharides from regenerated amorphous cellulose.

## Introduction

Lignocellulose-rich biomass has been recognized as a sustainable source to produce fuels, chemicals, and materials, and it will, eventually, contribute to the replacement of nonrenewable fossil-based products.^1^ A key step in this biorefinery concept is the degradation of abundantly present cell wall polysaccharides (i.e., cellulose and hemicellulose) into fermentable monomeric sugars.^2^ This widely studied process is optimal when using an enzymatic cocktail of (hemi-)cellulases and lytic polysaccharide monooxygenases (LPMOs).^3^ LPMOs are copper-dependent enzymes and currently classified into sequence-based “Auxiliary Activity” families AA9–11 and AA13–16 in the Carbohydrate-Active enZYmes (CAZy) database (http://www.cazy.org).^4^ In this study we focus on LPMOs from the largest AA family (i.e., AA9). So far, AA9 LPMOs are all fungal enzymes and active on cellulose.^5^ Although in the past decade much research has been conducted to disclose the catalytic mechanisms and structural features of AA9 LPMOs,^6^ insight into cellulose specificity (i.e., toward different cellulose types) and corresponding product profiles is still limited. Assigning such properties to individual LPMOs, and highlighting their specific product profiles, is a prerequisite to find the most appropriate candidates for envisaged applications.

AA9 LPMOs catalyze the hydroxylation of either C1-, C4-, or both C1- and C4-carbon positions (i.e., defining their regioselectivity) in cellulose using O_2_ and/or H_2_O_2_ as co-substrate and an external electron donor (e.g., ascorbic acid).^6^ Several studies have proposed that the regioselectivity for C1- or C4-oxidation depends on how LPMOs bind to their substrate.^7^ In addition, LPMOs can be connected to a carbohydrate-binding module (CBM), and, as suggested in several studies, this might influence the regioselectivity of oxidation.^5^ It has been reported that roughly one-fifth of AA9 LPMOs are fused to a C-terminal cellulose-specific CBM1 via a flexible linker.^8^

Apart from regioselectivity, AA9 LPMOs exhibit substrate specificity. Some AA9 LPMOs were reported to cleave xyloglucan, glucomannan, mixed β-(1→3, 1→4)-linked glucan, (cellulose-associated) xylan, and even soluble cello-oligosaccharides, all in addition to cellulose.^5^ To understand if substrate specificity correlates with AA9 LPMO structural elements surrounding the active sites, a structure-based multiple sequence alignment and a phylogenetic analysis have been performed for some AA9 LPMOs.^9^ Five segments surrounding the active site were linked to substrate recognition.^9^ Moreover, through this structure–function analysis, oxidative xyloglucan-active LPMOs, being tolerant or intolerant to xyloglucan substitutions, could be distinguished from cellulose-specific LPMOs.^10^ So far, details about the specificity of AA9 LPMOs toward various types of cellulose have remained elusive.

Cellulose is a homopolymer consisting of β-(1→4)-linked linear glucan chains.^11^ In plant cell walls, the linear glucan chains of cellulose are assembled via hydrogen bonds and van der Waals forces to form crystalline microfibrils.^11^ The chain length of cellulose can be expressed by the degree of polymerization (DP).^11^ Depending on the source, the treatment, and the assays used, the DP values of cellulose vary from 300 to 15 000.^12^ Commercial cellulose is usually extracted and purified from lignocellulose-rich biomass, and the most widely applied type is microcrystalline cellulose (i.e., Avicel PH-101 (AVI)).^13^ Microcrystalline cellulose can be used to prepare other cellulosic substrates with different properties (i.e., low crystallinity), such as phosphoric acid swollen cellulose (PASC) and regenerated amorphous cellulose (RAC).^14,15^ In addition, bacteria are known to synthesize a type of cellulose (e.g., bacterial cellulose (BC)) that differs from plant cell wall-derived cellulose in degree of polymerization, crystallinity, and morphology, as described elsewhere.^15,16^

As mentioned earlier, AA9 LPMO-cleavage profiles, or product profiles, of different types of cellulose have not been studied in detail. One of the main reasons is that most analytical techniques are only suitable to analyze soluble compounds and cannot be used to analyze insoluble cellulose directly. For that reason, the LPMO catalytic action toward cellulose has mainly been monitored with imaging techniques, for example, atomic force microscopy.^17^ Although this technique unravels interesting physical changes of the substrate, it does not provide data at the molecular level needed to study cleavage profiles.

In this study, we used three AA9 LPMOs from *Myceliophthora thermophila* C1 (*Mt*LPMOs) and one AA9 LPMO from *Neurospora crassa* (*Nc*LPMO9M), which differ in the presence or absence of CBM1, regioselectivity, and substrate specificity (Table S1). We analyzed their cellulose degradation profiles and detailed specificity toward AVI, RAC, and BC by quantifying the formed oxidized ends over time (in supernatant and residual cellulose) and profiling the soluble oxidized cello-oligosaccharides. We found that four AA9 LPMOs generated mainly oxidized cellobiose from BC, while more evenly distributed mixtures of oxidized cello-oligosaccharide (i.e., DP2–8) were observed for AVI and RAC. The cellulose specificity and product profiles of four AA9 LPMOs were modulated by the type of cellulose rather than being LPMO-type dependent.

## Experimental Section

### Carbohydrate Standards, Cellulose Type, and Other Chemicals

d-Glucose, d-gluconic acid (GlcOx^#^_1), and β-glucosidase from almond (9.3 U/mg, lyophilized powder) were purchased from Sigma-Aldrich (St. Louis, MO, U.S.A.). Ascorbic acid (Asc) was purchased from VWR International (Radnor, PA, U.S.A.). d-Cellobionic acid (GlcOx^#^_2) ammonium salt was purchased from Toronto Research Chemicals (Toronto, Ontario, Canada). Water used in all experiments was produced via a Milli-Q system (Millipore, Molsheim, France). Bacterial cellulose (BC) was produced by *Komagataeibacter xylinus* and prepared as described by Valenzuela et al.^15^ Regenerated amorphous cellulose (RAC) was prepared from AVI (Avicel PH-101, Sigma-Aldrich) as described previously.^18,19^ Other carbohydrates were purchased from either Sigma-Aldrich or Megazyme (Bray, Ireland). Cellulase cocktails of Celluclast 1.5 L and Accellerase BG were obtained from Novozymes (Bagsværd, Denmark) and Genencor (Palo Alto, CA, U.S.A.), respectively.

### Enzyme Production and Purification

The genes encoding *Mt*LPMO9B (*Mt*9B^+^, MYCTH_80312; UniProt ID, G2QCJ3) and the one with the truncated linker and CBM1 domain (*Mt*9B^–^) were homologously expressed in a low-protease/(hemi-)cellulose producing *Myceliophthora thermophila* C1 strain (IFF Nutrition & Biosciences, Leiden, The Netherlands), as described elsewhere.^20,21^*Mt*9B^+^ and *Mt*9B^–^ were purified in three subsequent chromatographic steps (see Supporting Information for more detail). The production and purification of *Mt*LPMO9I, *Mt*LPMO9H, and *Nc*LPMO9M are also described in the Supporting Information.

### Incubation of Different Cellulose Types with AA9 LPMOs

Three cellulose types (BC, AVI, and RAC) were suspended in 50 mM ammonium acetate buffer (pH 5.0) in the absence or presence of 1 mM ascorbic acid (Asc, final concentration). Subsequently, *Mt*9B^+^ and *Mt*9B^–^ were added to the corresponding mixtures at a final concentration of 0.75 μM. The time-course incubations (1, 2, 4, 6, 16, and 24 h) with BC and AVI were prepared in separate 2 mL Eppendorf tubes (Hamburg, Germany) with final volumes of 1 mL, while the time-course incubations with RAC were prepared in 15 mL Greiner tubes (Lake Forest, IL, U.S.A.) with final volumes of 3 mL. BC and AVI samples were incubated in the Eppendorf Thermomixer Comfort at 800 rpm placed in an almost vertical direction, and RAC samples were incubated in a head-over-tail disk rotator under 25 rpm at 30 or 50 °C. Incubations performed in either a thermomixer or in a head-over-tail disk rotator resulted in comparable outcomes (not shown). Control samples containing cellulose and enzymes either without Asc or with 1 mM Asc were incubated accordingly in the same way. At each time point, a 0.5 mL RAC sample was taken out from the tube, and BC/AVI samples were removed from the incubators. The incubation was stopped by the separation of supernatant (SUP) from the residue (RES) directly after centrifugation at 22 000 × *g* for 10 min at 4 °C. SUP and RES of all time points were stored at −20 °C for further analysis. Another batch of BC and RAC end-point (24 h) incubations with *Mt*LPMO9I, *Mt*LPMO9H, and *Nc*LPMO9M was performed in the same way (only at 30 °C) as described earlier. All incubations were performed in duplicate, and SUP was diluted 5 times prior to high-performance anion-exchange chromatography (HPAEC) analysis for oligosaccharide profiling.

### Quantification of Gluconic Acid and Cellobionic Acid in the Sample Supernatant

To investigate the amounts of C1-oxidized ends in the supernatant of the samples, (C1-oxidized) cello-oligosaccharides were hydrolyzed by β-glucosidase to GlcOx^#^_1 and GlcOx^#^_2, which were quantified by using HPAEC. β-Glucosidase hydrolysis was performed by following a previously described method with the following modifications.^22^ β-Glucosidase was first dissolved in 62.5 mM ammonium acetate (pH 5.0) buffer to give a 2.5 U/mL stock solution. Subsequently, 400 μL of β-glucosidase stock solution was mixed with 100 μL of SUP from each sample of time-course incubation of BC, AVI, and RAC with *Mt*9B^+^ or *Mt*9B^–^, as well as control samples, to a concentration of 1 U/mL. The reaction was incubated in an Eppendorf Thermomixer Comfort at 800 rpm at 37 °C for 24 h. The amounts of released GlcOx^#^_1 and GlcOx^#^_2 in SUP were quantified by using HPAEC with calibration curves of known concentrations (0–50 μg/mL each) of GlcOx^#^_1 and GlcOx^#^_2. β-Glucosidase-hydrolyzed samples were diluted 5 times for HPAEC analysis.

### Quantification of Gluconic Acid Released from Residual Cellulose

To investigate the amounts of C1-oxidized ends in the residual cellulose, RES was hydrolyzed by a commercial cellulase cocktail to GlcOx^#^_1, which was quantified by using HPAEC. A cellulase cocktail hydrolysis of residual cellulose was carried out based on a previously described method.^22^ Celluclast 1.5 L and Accellerase BG were fractionated by size-exclusion chromatography (SEC) to discard fractions with impurities that disturb the HPAEC quantification of gluconic acid. The fractionation of Celluclast 1.5 L and Accellerase BG is described in the Supporting Information. The cellulase cocktail stock solution was first prepared by mixing purified Celluclast 1.5 L (final concentration of 2.5 mg/mL; 1.25 mg protein/mg residue) and Accellerase BG (final concentration of 1 mg/mL; 0.5 mg protein/mg residue) with 50 mM ammonium acetate (pH 5.0) buffer. Subsequently, 500 μL of cellulase cocktail stock solution was mixed with RES from each sample of time-course incubation of BC, AVI, and RAC with *Mt*9B^+^ or *Mt*9B^–^, as well as control samples. Due to the removal of SUP after centrifugation and the addition of 500 μL of Asc-free cellulase cocktail stock solution, only trace amounts of Asc remained in RES, which is too low to drive the *Mt*9B^+^ and *Mt*9B^–^ reactions to confound the results of RES hydrolysis. The hydrolysis was incubated in an Eppendorf Thermomixer Comfort at 800 rpm at 50 °C for 48 h. The amount of released GlcOx^#^_1 in RES was quantified by using HPAEC with calibration curves of known concentrations (0–50 μg/mL) of GlcOx^#^_1 standard. Samples hydrolyzed by the cellulase cocktail were diluted 10 times prior to HPAEC analysis.

### HPAEC Analysis for Gluconic Acid and Cellobionic Acid Quantification and for Oligosaccharide Profiling

GlcOx^#^_1, GlcOx^#^_2, and (oxidized) cello-oligosaccharides were analyzed by HPAEC. The analysis was performed on an ICS-5000 system (Dionex, Sunnyvale, CA, U.S.A.) equipped with a CarboPac PA-1 column (2 mm i.d. × 250 mm; Dionex) in combination with a CarboPac PA guard column (2 mm i.d. × 50 mm; Dionex). The system was equipped with pulsed amperometric detection (PAD). Mobile phases were (A) 0.1 M NaOH and (B) 1 M NaOAc in 0.1 M NaOH. The column temperature was set to 20 °C, and two elution programs were used. For the quantification of GlcOx^#^_1 (and GlcOx^#^_2), a 35 min elution program was used as described previously.^22^

For profiling the (oxidized) cello-oligosaccharides, a 65 min elution profile was applied, as also described previously.^18,23^ After HPAEC-PAD profiling, the peak area of each DP of C1-oxidized cello-oligosaccharide present in SUP from all time-point incubations was manually integrated and recorded. Total peak area (calculated by the sum of all DPs) in each 24 h incubation sample was set as 100%, and the percentage of each DP of C1-oxidized cello-oligosaccharide in other time-course incubated samples was expressed accordingly.

## Results and Discussion

### Substrate Specificity Screening of *Mt*9B^+^ and *Mt*9B^–^

The substrate specificities of the purified *Mt*9B^+^ and *Mt*9B^–^ (Figure S1) were screened with a wide range of carbohydrates (the experimental setup is described in the Supporting Information), and results are shown in Table 1. *Mt*9B^+^ and *Mt*9B^–^ were free of cellulase side-activity, as shown in Figure S2. In the presence of Asc, *Mt*9B^+^ and *Mt*9B^–^ produced a range of detectable C1-oxidized cello-oligosaccharides from all four types of cellulose (with limited activity toward carboxymethyl cellulose; data not shown). Because both *Mt*9B^+^ and *Mt*9B^–^ released C1-oxidized cello-oligosaccharides, it was concluded that the CBM1 had no effect on the regioselectivity of oxidation of *Mt*LPMO9B. No activity of *Mt*9B^+^ or *Mt*9B^–^ was detected for any of the hemicellulosic substrates tested, not even in mixtures with RAC. Given the lack of activity on soluble cello-oligosaccharides, both *Mt*9B^+^ and *Mt*9B^–^ were concluded to be specifically active toward polymeric cellulosic structures. Similar results for *Mt*9B^+^ were described by Frommhagen et al.,^23^ although in the current study a more extensive substrate screening was performed.

**Table 1:** Carbohydrate Activity Screening of *Mt*9B^+^ and *Mt*9B^–^ in the Presence of Asc

Oxidized oligosaccharides observed (+) or not (−) (in the presence of 1 mM Asc)				
	*Mt*9B^**+**^		*Mt*9B^**–**^	
Substrate	GlcOx_n^a^	HemiOx_n^b^	GlcOx_n^a^	HemiOx_n^b^
Cellulose				
BC	+	–	+	–
AVI	+	–	+	–
RAC	+	–	+	–
Carboxymethyl cellulose	+	–	+	–
Hemicellulose				
Xyloglucan (tamarind)	–	–	–	–
β-Glucan (barley)	–	–	–	–
β-Glucan (oat spelt)	–	–	–	–
Xylan (oat spelt)	–	–	–	–
Xylan (birchwood)	–	–	–	–
Arabinoxylan (wheat)	–	–	–	–
Mannan (acacia)	–	–	–	–
Galactan (potato)	–	–	–	–
Glucomannan (konjac)	–	–	–	–
Arabinan (sugar beet)	–	–	–	–
Laminarin (*Laminaria digitata*)	–	–	–	–
RAC/hemicellulose combination				
RAC + xyloglucan (tamarind)	+	–	+	–
RAC + xylan (birchwood)	+	–	+	–
Oligosaccharides				
Cello-oligosaccharides (DP2–6)	–	–	–	–
Xylo-oligosaccharides (DP2–6)	–	–	–	–

a GlcOx_n: oxidized cello-oligosaccharides.

b HemiOx_n: oxidized hemicello-oligosaccharides from the corresponding hemicellulose.

The influence of CBM1 on the regioselectivity of oxidation has previously been investigated for several AA9 LPMOs.^17,24−26^ Laurent and Sun et al. and Danneels et al. reported that the removal of a CBM1 from *Nc*LPMO9C and *Hj*LPMO9A did not alter their regioselectivity toward cellulose.^9,25^ In another study, although the regioselectivity was not changed, the ratio between C1- and C4-oxidized cello-oligosaccharides released by *Pa*LPMO9H with and without CBM1 was different.^17^ Little is known about the influence of the presence of a CBM on the substrate specificity of AA9 LPMOs. Only for *Nc*LPMO9C was it reported that the substrate specificity did not change after the truncation of its CBM1.^24^ Thus, more detailed characterization is required for a better understanding of the catalytic performance of AA9 LPMOs with different cellulose substrates.

### CBM1 Promoted *Mt*LPMO9B Cellulose Degradation

As *Mt*9B^+^ or *Mt*9B^–^ showed only oxidative cleavage of different cellulose types, we further investigated their binding affinity (the experimental setup is described in the Supporting Information) and oxidative cleavage toward BC, AVI, and RAC. As shown in Table S2, the amount of cellulose-bound *Mt*9B^+^ was higher compared to *Mt*9B^–^ in all BC, AVI, and RAC samples at both 30 and 50 °C. This is in line with other LPMO studies where the presence of CBM1 resulted in more protein binding per gram of substrate.^9,27−30^

To quantify the oxidative cleavage of *Mt*9B^+^ or *Mt*9B^–^ over time, soluble (C1-oxidized) cello-oligosaccharides in SUP and the insoluble (oxidized) cellulose in RES were hydrolyzed by β-glucosidase and a cellulase cocktail, respectively. Subsequently, GlcOx^#^_1 and GlcOx^#^_2 were quantified to indicate the level of oxidation. A previous study from our laboratory showed that both GlcOx^#^_1 and GlcOx^#^_2 were released from cello-oligosaccharides by β-glucosidase and that only GlcOx^#^_1 was released by the cellulase cocktail.^22^ In addition, full hydrolysis of RES from BC and RAC was achieved. Although only ∼67% of AVI was hydrolyzed under the same conditions (Figure S3), it is still possible to compare the results of AVI hydrolysis to the results obtained from BC and RAC hydrolysis.

**Figure 1:**
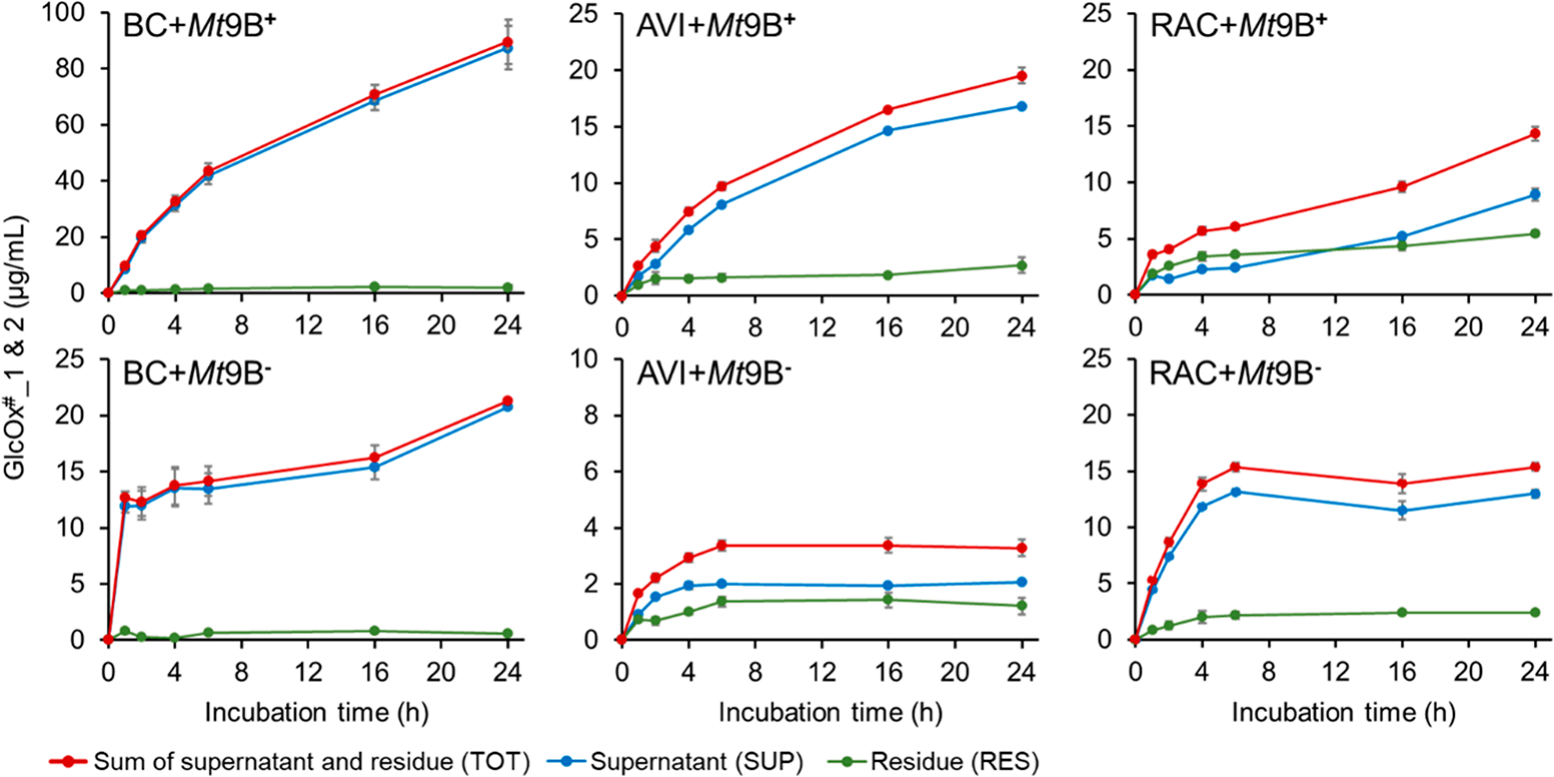
Amounts of gluconic acid (GlcOx^#^_1) and cellobionic acid (GlcOx^#^_2) generated from BC, AVI, and RAC by *Mt*9B^+^ and *Mt*9B^–^ after subsequent hydrolysis, in supernatant (SUP, blue), residue (RES, green), and the sum of both (TOT, red) over time at 30 °C. Error bars (gray) indicate the standard deviations (± std) of duplicate incubations. Amounts of GlcOx^#^_1 and GlcOx^#^_2 generated from BC, AVI, and RAC by *Mt*9B^+^ and *Mt*9B^–^ after subsequent hydrolysis over time at 50 °C are shown in Figure S4.

The quantifications of the time-dependent oxidative cleavage of the different cellulose types by *Mt*9B^+^ or *Mt*9B^–^ are shown in Figure 1 and Figure S4 for incubations at 30 and 50 °C, respectively. At 30 °C (Figure 1 and Table S3), *Mt*9B^+^ produced a much higher amount of GlcOx^#^_1 and 2 from BC (up to ∼89 μg/mL) and AVI (up to ∼19 μg/mL) compared to the amount released by *Mt*9B^–^ (∼21 μg/mL from BC and ∼3 μg/mL from AVI) in 24 h. For the RAC digests, *Mt*9B^–^ released higher amounts of oxidized products than *Mt*9B^+^ in the early time points (until 6 h) at 30 °C. After 6 h, *Mt*9B^–^ did not release more GlcOx^#^_1 and 2, while *Mt*9B^+^ continued generating GlcOx^#^_1 and 2. At 50 °C (Figure S4 and Table S4), *Mt*9B^–^ almost stopped releasing more GlcOx^#^_1 and 2 from all BC, AVI, and RAC after 4 h, while *Mt*9B^+^ still generated GlcOx^#^_1 and 2 (except AVI). Nevertheless, at 50 °C, the total amount of GlcOx^#^_1 and 2 in the *Mt*9B^+^ samples (∼86 μg/mL from BC, ∼6 μg/mL from AVI, and ∼56 μg/mL from RAC) was much higher compared to the total amount in *Mt*9B^–^ samples (∼18 μg/mL from BC, ∼1 μg/mL from AVI, and ∼8 μg/mL from RAC), and after 24 h the difference was even larger than at 30 °C.

In summary, the oxidative cleavage by *Mt*LPMO9B toward cellulose was modulated and influenced by the type of cellulose, CBM1, and temperature. Overall, the amount of GlcOx^#^_1 and 2 differed between the three types of cellulose, hinting at a different cellulose-specific behavior. This result will be discussed further later. At an elevated temperature (50 °C), the cellulose specificity was still observed; however, on the basis of the observation that oxidative cleavage stopped, the inactivation of *Mt*LPMO9B was more pronounced compared to incubations at 30 °C. This was particularly striking for *Mt*9B^–^ lacking CBM1. In addition, a larger difference in the amount of oxidized products obtained by *Mt*9B^+^ and *Mt*9B^–^ was observed after summation of the amounts in SUP and RES at 50 °C compared to that at 30 °C. This further suggests that the CBM1 might stabilize or “help” the *Mt*LPMO9B to act more pronounced and reduce the inactivation at an elevated temperature. A similar suggestion was reported for *Bc*LPMO10A, a bacterial LPMO, for which it has been shown that the removal of its CBM5 leads to fast enzyme inactivation and, thus, a decrease of oxidative cleavage.^31^ In addition to stabilization, it has been reported that CBM-driven substrate binding concentrates the LPMO on the substrate, which might further explain the observed higher amount of degradation products for *Mt*9B^+^ compared to *Mt*9B^–^.^26−30,32^

Next, on the basis of the results shown in Figure 1, to further investigate the influence of cellulose types on the oxidized products released by LPMOs, the amounts of GlcOx^#^_1 and 2 present in SUP were calculated as percentages (%-Ox) of the total amount (TOT). This was done in order to mimic the parameter “percentage of soluble reducing sugar (%-Sugar)” used to describe the catalytic performance of cellulases in a filter paper assay.^33−39^ For both *Mt*9B^+^ and *Mt*9B^–^, the %-Ox was >95% in the BC digests at 30 and 50 °C. However, the %-Ox decreased to approximately 85% and 62% in *Mt*9B^+^-AVI and -RAC digests (30 °C, 24 h), respectively, while at 50 °C it changed to approximately 75% and 80%, respectively. Likewise, in *Mt*9B^–^-AVI and -RAC digests, the %-Ox decreased to approximately 65% and 82% (30 °C, 24 h), respectively, while at 50 °C *Mt*9B^–^-AVI and -RAC digests were not representative due to the early inactivated *Mt*9B^–^. The difference of %-Ox in *Mt*9B^+^-AVI and -RAC and *Mt*9B^–^-AVI and -RAC digests might relate to a more pronounced binding of CBM1 to crystalline cellulose than to RAC.^40,41^ Courtade et al. observed a higher fraction of %-Ox in a full-length *Sc*LPMO10C-AVI digest compared to a CBM-truncated *Sc*AA10-AVI digest (at comparable substrate concentrations), which has been explained by the immobilizing effect of the CBM.^28^ This effect, as suggested by the authors, could keep the LPMO catalytic domain in a certain cellulose area and thereby increase the chance for two (or more) cuts in the same cellulose chain.^28^ Indeed, the higher %-Ox from *Mt*9B^+^-AVI digest than from *Mt*9B^–^-AVI digest might result from such an immobilizing effect of CBM1. However, in contrast, the product profiles obtained were similar for *Mt*9B^+^ and *Mt*9B^–^, and they are further discussed in the later sections.

For cellulases, exo-cleavage and a processive catalytic action are considered if the parameter %-Sugar is >90%, while 50%–70% reflects more endocleavage and random-like action.^35−37,42^ Because of their distinct structure and catalytic mechanisms compared to cellulases, LPMOs are not expected to act in a processive manner. However, the different %-Ox from BC, AVI, and RAC still indicate that the catalytic performance of *Mt*LPMO9B (both *Mt*9B^+^ and *Mt*9B^–^) is modulated by the type of cellulose. To gain more insight into the mode of cleavage of the three types of cellulose, corresponding product profiles were studied in detail.

### Distinct Product Profiles of Oxidized Cello-Oligosaccharide Released from Different Cellulose Types

**Figure 2:**
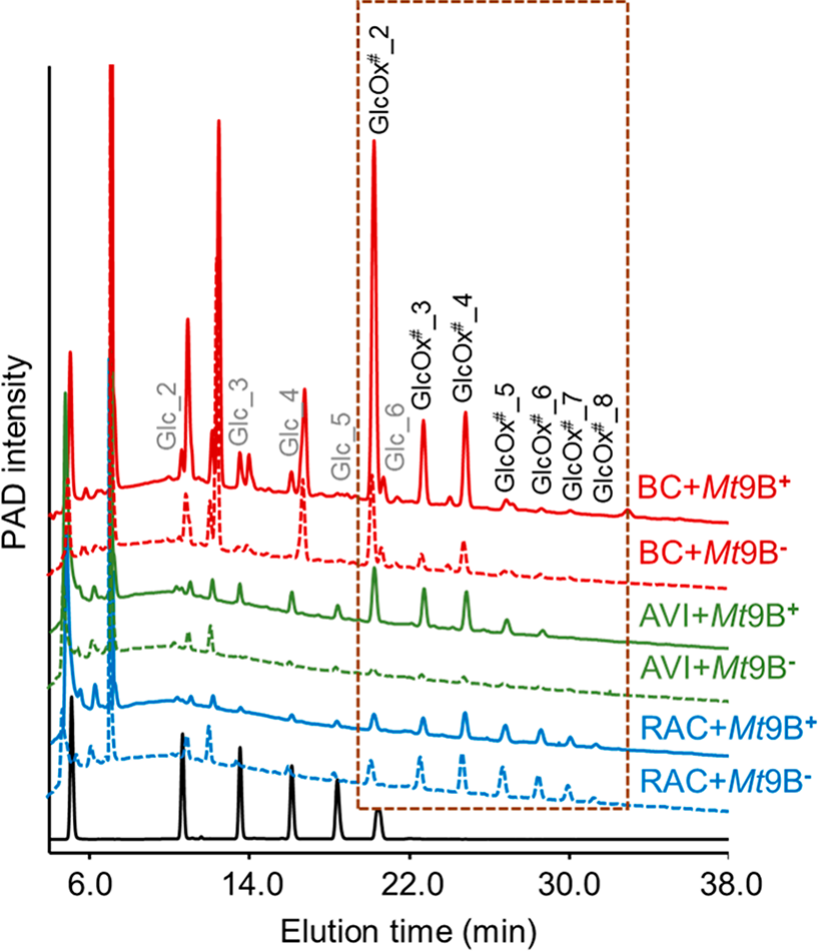
HPAEC elution patterns of supernatants (SUP) from BC, AVI, and RAC digests (24 h incubation) of *Mt*9B^+^ and *Mt*9B^–^ in the presence of Asc at 30 °C. HPAEC elution patterns of supernatants generated at 50 °C are shown in Figure S5. An HPAEC chromatogram from one of the duplicate samples is shown here, as they are identical. Annotation of non- (Glc_2–Glc_6) and C1-oxidized (GlcOx^#^_2–GlcOx^#^_8) cello-oligosaccharides is based on a previous study.^18,23^ In this study, C1-oxidized cello-oligosaccharides are the most relevant, and their elution range is located in the brown frame. Because of the presence of the carboxylic acid end in the C1-oxidized cello-oligosaccharides, they bind stronger to the HPAEC column and thus are eluted later in HPAEC compared to non-oxidized cello-oligosaccharides. A standard containing a mixture of Glc_1–Glc_6 (from left to right in the chromatogram) is shown in black. The SUP of the control incubations is shown in Figure S2.

Soluble oxidized cello-oligosaccharides formed at 24 h in BC, AVI, and RAC (30 and 50 °C) were analyzed by HPAEC-PAD, and the corresponding chromatograms are shown in Figure 2 and Figure S5. Overall, non- and C1-oxidized cello-oligosaccharides (GlcOx^#^_n) were detected in the incubations of all three cellulose types with *Mt*9B^+^ and *Mt*9B^–^. However, the product profiles of C1-oxidized cello-oligosaccharides were different among BC, AV,I and RAC samples. In both *Mt*9B^+^- and *Mt*9B^–^-BC samples, GlcOx^#^_2 was the most pronounced followed by GlcOx^#^_3 and GlcOx^#^_4, at both 30 and 50 °C (Figure 2 and Figure S5). Only very low amounts of GlcOx^#^_5–GlcOx^#^_8 were detected, indicating that both *Mt*9B^+^ and *Mt*9B^–^ formed mainly short oligosaccharides from BC. For the *Mt*9B^+^-AVI samples (Figure 2 and Figure S5), again mainly GlcOx^#^_2–GlcOx^#^_4 were formed, but the ratio between GlcOx^#^_5–GlcOx^#^_8 and GlcOx^#^_2–GlcOx^#^_4 increased. At 30 and 50 °C, the amounts of oxidized cello-oligosaccharides in the *Mt*9B^–^-AVI sample were too low to see clear patterns. In comparison to the BC and AVI samples, a more even distribution pattern of GlcOx^#^_2–GlcOx^#^_8 products was observed in *Mt*9B^+^- and *Mt*9B^–^-RAC samples.

**Figure 3:**
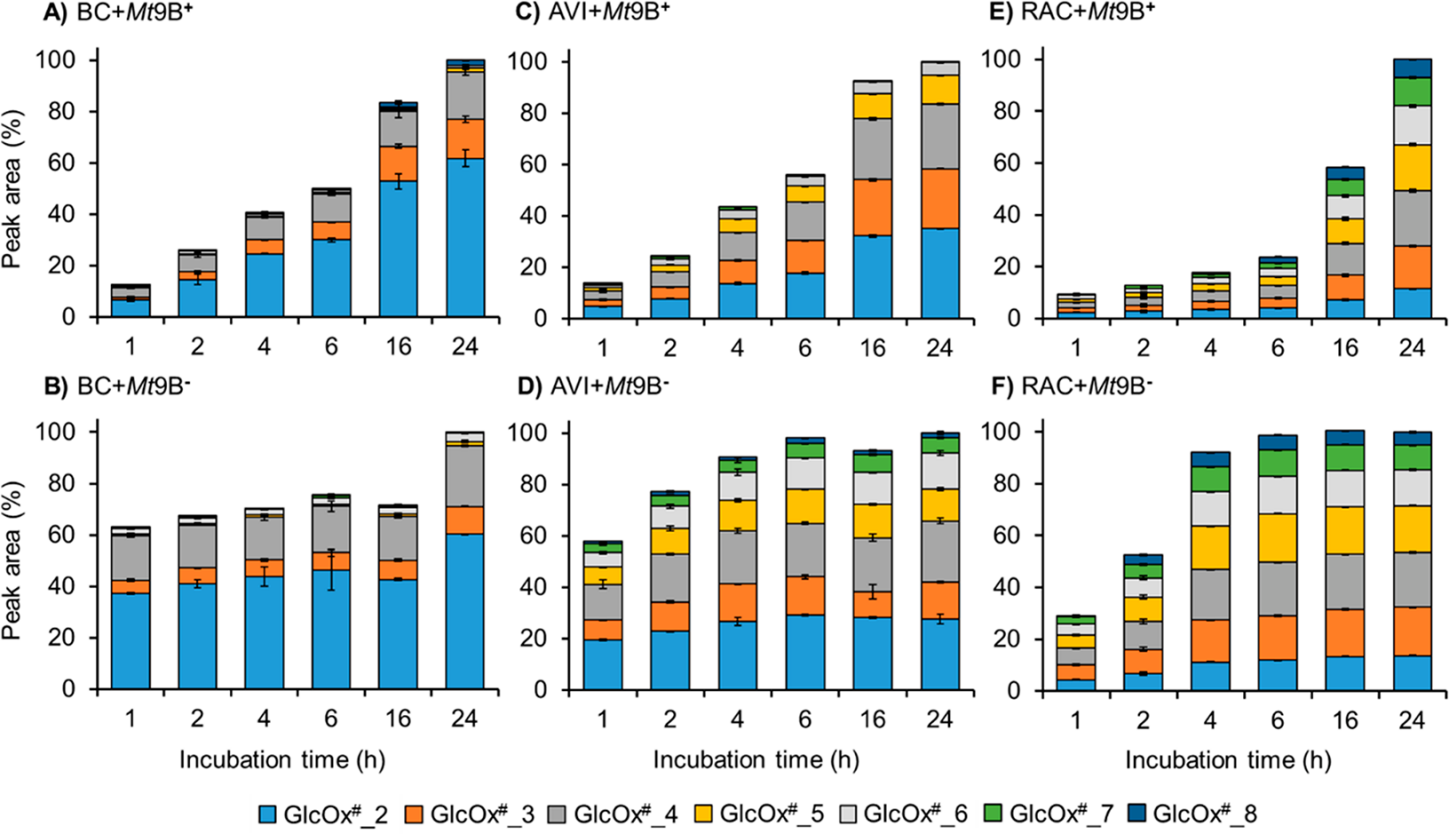
Relative quantification of peak area of each DP of oxidized cello-oligosaccharides (GlcOx^#^_2–GlcOx^#^_8) generated by *Mt*9B^+^ and *Mt*9B^–^ from the time-course incubation with BC, AVI, and RAC at 30 °C. The total peak area of each 24 h sample was set to 100%. The relative quantification of the peak area of released oxidized cello-oligosaccharides at 50 °C is shown in Figure S6. Error bars for each DP of oxidized cello-oligosaccharides indicate the standard deviations (± std) of duplicate incubations.

To further investigate the product profiles of BC, AVI, and RAC digests, we quantified each DP of oxidized cello-oligosaccharides formed over time. Due to the lack of GlcOx^#^_3–GlcOx^#^_8 standards, quantification was based on the peak area of each DP and expressed as the percentage of total peak area of oxidized cello-oligosaccharides from the corresponding 24 h sample (Figure 3 and Figure S6). In line with the previously described patterns, both *Mt*9B^+^ and *Mt*9B^–^ predominantly released GlcOx^#^_2 (>60%) followed by GlcOx^#^_4 (∼20%) and GlcOx^#^_3 (∼10–15%) from BC over time at 30 °C (Figure 3).

In the AVI samples, GlcOx^#^_2 and GlcOx^#^_4 reflected the main products; however, the proportion of larger GlcOx^#^ products was higher than that in the BC samples (Figure 3). In the RAC samples, the percentages of GlcOx^#^_2 and GlcOx^#^_4 were the lowest at all time points (Figure 3). Additionally, compared to the BC and AVI samples, the proportion of higher DP of oxidized cello-oligosaccharides (DP5–8) increased in the RAC samples, while the AVI-based product profiles represent an intermediate situation. As described in the previous section, the immobilizing effect of a CBM is also expected to result in smaller oxidized products, as shown by the higher percentage of GlcOx^#^_2–GlcOx^#^_4 in the full-length *Sc*LPMO10-AVI digest compared to the CBM-truncated *Sc*AA10-AVI digest.^28^ In that study the authors suggest that, when a CBM is present, the chance of multiple cleavages in the same cellulose chain is higher, and thus, shorter oxidized cello-oligosaccharides can be expected.^28^ However, our data do not show such difference in product profiles for the full-length and CBM-truncated LPMO used, and hence, we cannot conclude that the product profiles are CBM-dependent.

These distinct profiles from BC, AVI, and RAC samples at 30 °C were found to be similar in the BC, AVI, and RAC digests with *Mt*9B^+^ and *Mt*9B^–^ at 50 °C (Figure S6), although the amounts of each DP were different compared to the samples at 30 °C.

**Figure 4:**
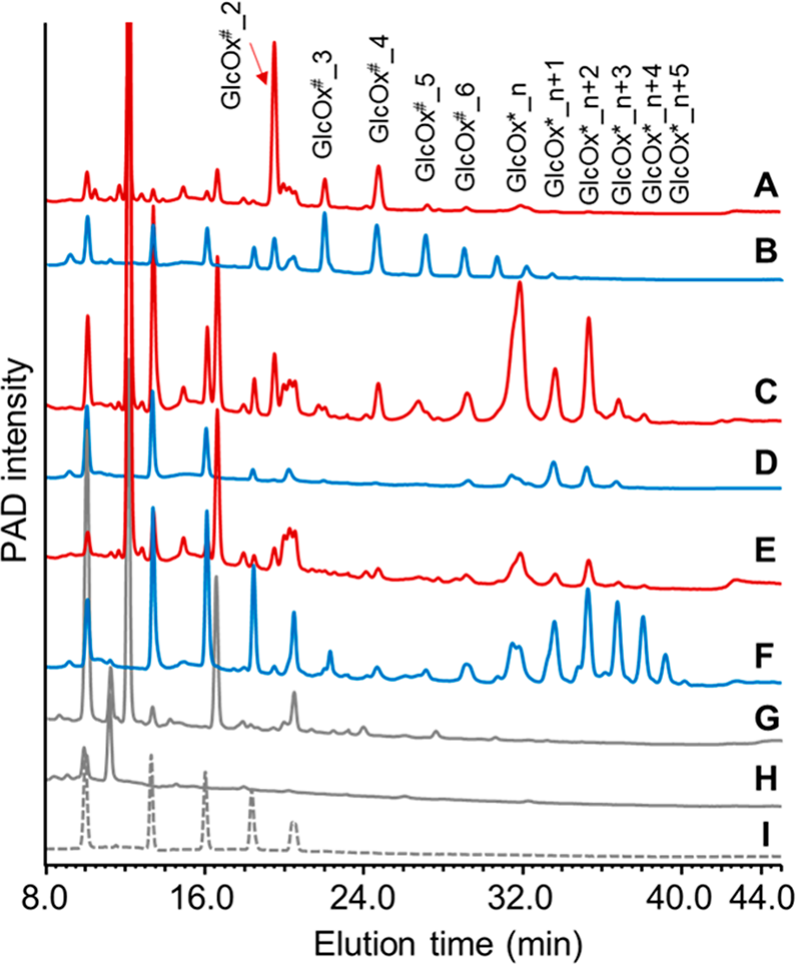
HPAEC elution patterns of supernatants (SUP) from the incubation of BC (red) and RAC (blue) with *Mt*LPMO9I (A, B), *Mt*LPMO9H (C, D), and *Nc*LPMO9M (E, F). SUP of control incubations (BC + Asc (G) and RAC + Asc (H)) and cello-oligosaccharide standard (I) are shown in gray solid lines and a dashed line, respectively.

To further substantiate that the mode of cleavage may relate to the type of cellulose, rather than to the type of LPMO, cellulose digests of three other AA9 LPMOs were compared to those from *Mt*LPMO9B. These three others (Table S1) were the previously characterized C1-oxidizing *Mt*LPMO9I (no CBM),^18^ C1-/C4-oxidizing *Mt*LPMO9H (having a CBM1),^43^ and C1-/C4-oxidizing *Nc*LPMO9M (no CBM).^44^ BC and RAC digests with these three LPMOs (24 h and 30 °C) were analyzed by HPAEC (Figure 4). Similar to the product profiles in *Mt*LPMO9B-BC digests, *Mt*LPMO9I generated mainly short oxidized cello-oligosaccharides (DP2–4) from BC, while all DPs of oxidized cello-oligosaccharides were present in a more evenly distributed pattern in the RAC sample (Figure 4). In *Mt*LPMO9H- and *Nc*LPMO9M-BC digests, short (C4-oxidized) cello-oligosaccharides (GlcOx*_n–GlcOx*_n+2) were predominant. Again, more even distribution profiles (of C4-oxidized cello-oligosaccharides) were seen in their RAC digests. The concentrations of C1-oxidized products released by *Mt*LPMO9H and *Nc*LPMO9M were too low to observe a clear cleavage pattern. These results indicate that not only *Mt*LPMO9B (with and without a CBM1) but also other AA9 LPMOs generate distinct cellulose degradation product profiles ranging from mainly oxidized cellobiose toward BC to a more even distribution toward RAC. In addition, estimated from their peak area, *Mt*LPMO9I and *Nc*LPMO9M released the highest quantities of oxidized products from RAC, whereas CBM1-containing *Mt*LPMO9H released the most oxidized products from BC.

### Proposed Scenario of *Mt*LPMO9B in Degrading Different Cellulose Types

As described earlier, the AA9 LPMO cellulose degradation profiles were mainly dependent on the type of cellulose used. BC, AVI, and RAC have been reported to vary in surface area, crystallinity, DP, and three-dimensional structure.^13,16,45−53^ In general, BC and AVI have a similarly high crystallinity;^15,16,45^ the crystallinity indices (CrI (%)) of BC and AVI were determined as 95.7 ± 0.5 and 92.7 ± 1.1, respectively.^15^ RAC has been shown to be completely amorphous due to the high concentration of phosphoric acid (86.2% wt/v) used to produce RAC.^14^ In a previous study, the CrI (%) of RAC was determined in a dried state (67.4 ± 1.6), explaining the rather high value obtained (i.e., due to recrystallization during the drying process).^15^ Compared to AVI, BC has a larger surface area and, therefore, has a higher accessibility.^16,45,54,55^ Further, BC consists of long ribbon-like microfibers with DPs ranging from 2000 to 6000, while AVI microfibers are shorter and thicker with a much lower DP (100–300).^13,16,49−53^ In addition, BC resembles a more well-arranged network compared to AVI.^15,16,45^ Gromovykh et al. suggested that the BC network forms three-dimensional layers of hollow cylinders, and each layer turns a small angle.^56^ For RAC, no typical DP lengths have been reported, but it can be expected that the DPs of RAC chains are shorter and more exposed compared to AVI seen in the process conditions (e.g., use of phosphoric acid) to produce RAC from AVI.^14,57^

**Figure 5:**
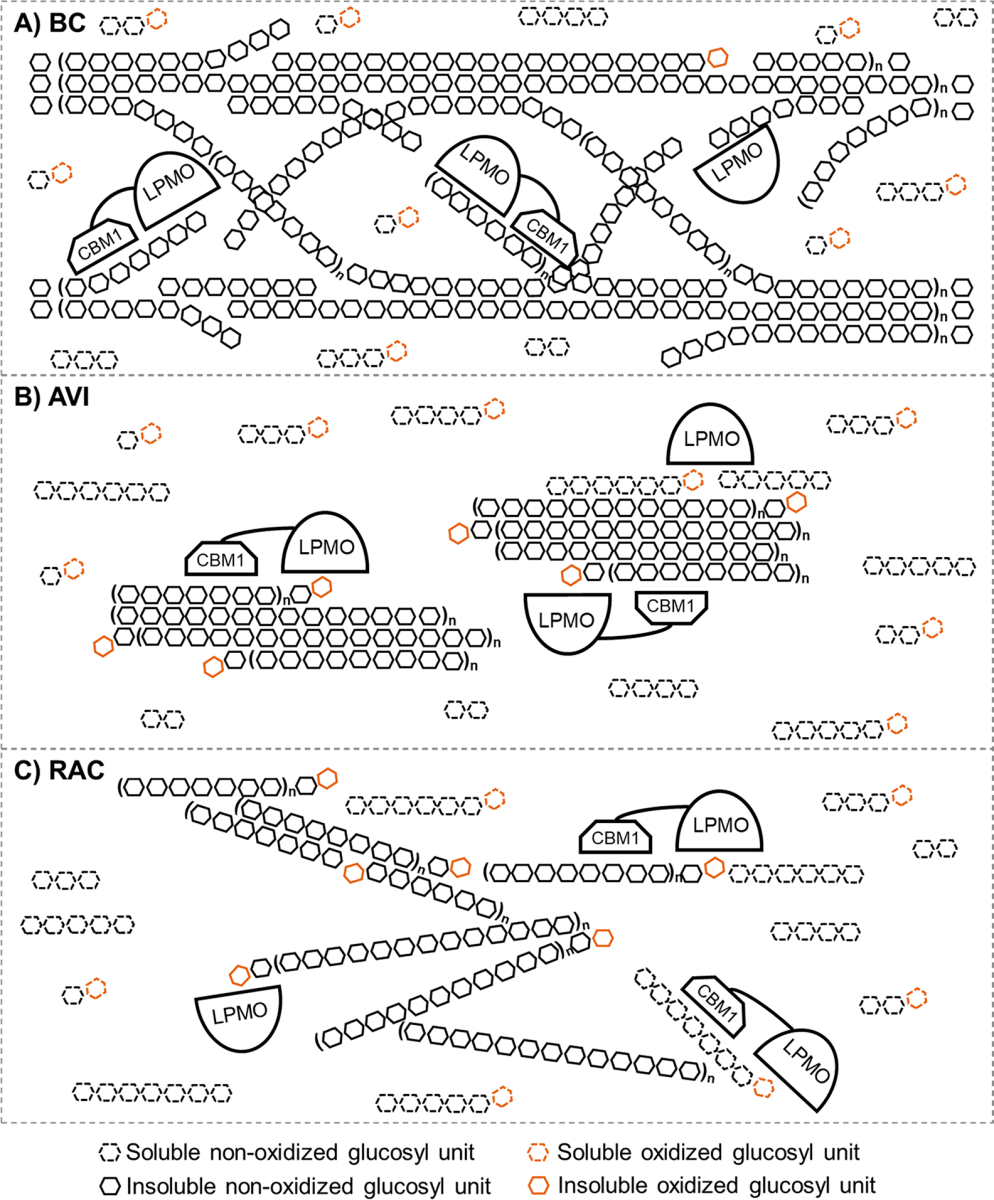
Schematic representation of the proposed scenario of *Mt*LPMO9B oxidative cleavage toward BC (A), AVI (B), and RAC (C). “n” indicates the number of repeating units. It should be noted that this figure presents a generic and schematic representation in two dimensions and might oversimplify how LPMOs cleave within cellulose chains.

On the basis of the distinct characteristics of BC, AVI, and RAC, together with our results, we propose different scenarios of how *Mt*LPMO9B oxidatively cleaves various cellulose types, as schematically depicted in Figure 5. On the basis of the model suggested by Gromovykh et al.^56^ we propose that *Mt*LPMO9B mainly cleaves the “connecting” region between layers. In between layers more chain ends can be expected, compared to the “layer” region, which might explain the pronounced formation of (oxidized) cellobiose (Figure 5A). This scenario corresponds to the product profiles found in the BC digests, mainly reflecting the formation of oxidized DP2–4 cello-oligosaccharides (oxidized cellobiose >60%) (Figures 2–4) and a %-Ox of >95% (Figure 1). Next, we propose a scenario of the *Mt*LPMO9B toward AVI (Figure 5B). Because of the lower homogeneity and shorter chain lengths compared to BC, the *Mt*LPMO9B has a lower chance to stay active on AVI, resulting in the formation of larger DPs of oxidized cello-oligosaccharides (Figures 2–4) and a %-Ox of 60%–80% (Figure 1). Still, oxidized DP2–4 cello-oligosaccharides were most pronounced in these AVI digests (Figures 2–4). For the amorphous and homogeneous RAC with more exposed glucan chains, we suggest that the LPMO has more chance to cleave in the middle of the RAC chains compared to the packed fibrous structures of BC and AVI (Figure 5C). Because of the lower DP of RAC compared to the DP of AVI, the polymeric cellulose chain more easily becomes soluble. This would explain why a more evenly distributed oxidized cello-oligosaccharide profile (Figures 2–4) is observed from the RAC digests compared to the BC and AVI digests.

The proposed scenario is mainly based on the quantification data of oxidized ends, oxidized cello-oligosaccharide profiles, and morphological properties of the used cellulose types. Other factors, for example, how long LPMOs (i.e., with or without a CBM) stay on the substrate, are also important to consider but are not included in our model.

## Conclusions

In this study, we compared several AA9 LPMOs for their reactivity with different types of cellulose and found that the substrate specificity and regioselectivity of the cleavage site were not altered by the presence of a CBM1. We also found that the CBM1 increased the release of oxidized cello-oligosaccharides by *Mt*LPMO9B, especially at the elevated temperature. This increased release corresponded to an increased binding affinity toward the substrates due to the presence of CBM1. Intriguingly, the length of the released cello-oligosaccharide was dependent on the characteristics of the cellulose type. From BC, mainly oxidized cellobiose was released regardless of the presence of CBM1, while from RAC and AVI, a more evenly distributed mixture of oxidized cello-oligosaccharides (DP2–8) was obtained. Our study highlights the importance of considering biopolymeric substrate characteristics when cleavage profiles and kinetics of AA9 LPMOs are studied.

## Supporting Information Available

Supplementary methods; SDS-PAGE analysis of purified *Mt*9B^+^ and *Mt*9B^–^; HPAEC elution patterns of control samples; percentages of hydrolysis of BC, AVI, and RAC by the fractionated cellulase cocktail; amounts of gluconic acid and cellobionic acid generated from BC, AVI, and RAC by *Mt*9B^+^ and *Mt*9B^–^ at 50 °C; HPAEC elution patterns of supernatants from different cellulose digests by *Mt*9B^+^ and *Mt*9B^–^ at 50 °C; relative quantification of peak area of oxidized cello-oligosaccharides released by *Mt*9B^+^ and *Mt*9B^–^ at 50 °C; four AA9 LPMOs used in this study; amounts of bound *Mt*9B^+^ and *Mt*9B^–^ on BC, AVI, and RAC; amounts of gluconic acid and cellobionic acid generated from BC, AVI, and RAC by *Mt*9B^+^ and *Mt*9B^–^ at 30 °C; and amounts of gluconic acid and cellobionic acid generated from BC, AVI, and RAC by *Mt*9B^+^ and *Mt*9B^–^ at 50 °C (PDF)
