# Supplementary material for: Oxidized Product Profiles of AA9 Lytic Polysaccharide Monooxygenases Depend on the Type of Cellulose
Source: ACS Sustain Chem Eng. 2021 Oct 13;9(42):14124–33. doi: 10.1021/acssuschemeng.1c04100 (PMC8549066; doi:10.1021/acssuschemeng.1c04100)
Supplement: Supplementary file 1 — sc1c04100_si_001.pdf [file sc1c04100_si_001.pdf]

Supporting Information

Oxidized Product Profiles of AA9 Lytic Polysaccharide Monooxygenases Depend on the Type of Cellulose

Peicheng Sun[a], Susana V. Valenzuela[b,c], Pimvisuth Chunkrua[a], Francisco. I. Javier Pastor[b,c], Christophe V. F. P. Laurent[d,e], Roland Ludwig[d], Willem J. H. van Berkel[a], Mirjam A. Kabel[a,*]

[a]Laboratory of Food Chemistry, Wageningen University & Research, Bornse Weilanden 9, 6708 WG Wageningen, The Netherlands

[b]Department of Genetics, Microbiology and Statistics, Faculty of Biology, University of Barcelona, Av. Diagonal 643, 08028 Barcelona, Spain

[c]Institute of Nanoscience and Nanotechnology (IN2UB), University of Barcelona, Av. Diagonal 645, 08028 Barcelona, Spain

[d]Biocatalysis and Biosensing Laboratory, Department of Food Science and Technology, BOKU–University of Natural Resources and Life Sciences, Vienna, Muthgasse 18, 1190 Vienna, Austria

[e]Institute of Molecular Modelling and Simulation, Department of Material Sciences and Process Engineering, BOKU–University of Natural Resources and Life Sciences, Vienna, Muthgasse 18, 1190 Vienna, Austria

*Corresponding author (Email: mirjam.kabel@wur.nl)

Number of pages: 17

Number of figures: 6

Number of tables: 4

Supporting Information contents:

Supplementary Methods

Figure S1. SDS-PAGE analysis of purified *Mt*9B$^+$ and *Mt*9B$^-$

Figure S2. HPAEC elution patterns of control samples

Figure S3. Percentage of hydrolysis of BC, AVI and RAC by the fractionated cellulase cocktail

Figure S4. Amounts of gluconic acid (GlcOx$^\#$_1) and cellobionic acid (GlcOx$^\#$_2) generated from BC, AVI and RAC by *Mt*9B$^+$ and *Mt*9B$^-$ after subsequent hydrolysis over time at 50 °C

Figure S5. HPAEC elution patterns of supernatants from different cellulose digests by *Mt*9B$^+$ and *Mt*9B$^-$ at 50 °C

Figure S6. Relative quantification of peak area of oxidized cello-oligosaccharides released by *Mt*9B$^+$ and *Mt*9B$^-$ at 50 °C

Table S1. Four AA9 LPMOs used in this study

Table S2. Amounts of bound *Mt*9B$^+$ and *Mt*9B$^-$ on BC, AVI and RAC

Table S3. Amounts of gluconic acid and cellobionic acid (GlcOx$^\#$_1 & 2, µg/mL) generated from BC, AVI and RAC by *Mt*9B$^+$ and *Mt*9B$^-$ after subsequent hydrolysis, in SUP, RES and TOT over time at 30 °C

Table S4. Amounts of gluconic acid and cellobionic acid (GlcOx$^\#$_1 & 2, µg/mL) generated from BC, AVI and RAC by *Mt*9B$^+$ and *Mt*9B$^-$ after subsequent hydrolysis, in SUP, RES and TOT over time at 50 °C

Supplementary Methods

Purification of *Mt*9B$^+$ and *Mt*9B$^-$

*Mt*9B$^+$ and *Mt*9B$^-$ were purified in three subsequent chromatographic steps. The initial anion exchange chromatography (AEC) and cation exchange chromatography (CEC) steps were the same as described previously.[1] The final CEC was performed with the following modifications. *Mt*9B$^+$ and *Mt*9B$^-$ obtained after the first CEC step were diluted two times with water before subjecting them to a Resource S column (30 × 16 mm internal diameter, GE Healthcare, Uppsala, Sweden) using an ÄKTA-Micro chromatography system (GE Healthcare). The column was pre-equilibrated with 10 mM sodium acetate buffer (pH 5.0). By using a flow rate of 1 mL/min, the unbound fraction was washed (three column volumes) and elution was performed by using a linear gradient from 0 to 0.5 M NaCl in 10 mM sodium acetate buffer pH 5.0 over one column volume. All unbound and eluted fractions (size 1 mL) were collected and immediately stored on ice. Peak fractions (based on UV 280 nm) were adjusted to a concentration of 2 mg/mL (determined by using Pierce™ BCA Protein Assay Kit (Sigma-Aldrich) and analyzed by sodium dodecyl sulfate-polyacrylamide gel electrophoresis (SDS-PAGE) as described previously.[2] The fractions containing either pure *Mt*9B$^+$ or pure *Mt*9B$^-$ were used for enzyme incubations.

Production and Purification of *Mt*LPMO9I, *Mt*LPMO9H and *Nc*LPMO9M

The genes encoding *Mt*LPMO9I (MTCTH_2299721, UniProt ID: G2Q774) and *Mt*LPMO9H (MYCTH_46583, UniProt ID: G2Q9T3) were homologously expressed in a low protease/low (hemi-) cellulose producing *Myceliophthora thermophila* C1 strain (IFF Nutrition & Biosciences, Leiden, The Netherlands), as described elsewhere.[3, 4] The purification of *Mt*LPMO9I has been described previously.[5]

*Mt*LPMO9H was purified by three subsequent chromatographic steps. Crude *Mt*LPMO9H-rich fermentation broth was filtrated and dialyzed against 10 mM potassium phosphate buffer pH 7.6 before chromatographic purification. The dialyzed *Mt*LPMO9H was purified by AEC followed by size exclusion chromatography (SEC). Purification settings and elution program of AEC and SEC have been described previously.[5] The SEC-purified *Mt*LPMO9H-containing fractions were further purified by CEC on an ÄKTA-Micro preparative chromatography system (GE Healthcare). *Mt*LPMO9H-containing fractions were loaded on a Resource Q column (30 × 16 mm internal diameter, GE Healthcare) pre-equilibrated with 10 mM sodium acetate buffer pH 4.5 (eluent A). The unbound fraction was firstly washed (one column

volume). Eluent B was 10 mM sodium acetate buffer (pH 4.5) containing 500 mM NaCl (eluent B). Elution (flow rate of 1 mL/min) was performed as follows: from 0% to 25% B in two column volumes; 25% B for one column volume; next 25% to 100% B over two column volumes and finally 100% B for four column volumes. All fractions were collected and immediately stored on ice. Peak fractions (based on UV 280 nm) were adjusted to an approximate concentration of 2 mg/mL (determined by BCA assay and analyzed by SDS-PAGE, as described previously[2]) to determine the *Mt*LPMO9H fractions. CEC-purified *Mt*LPMO9H-containing fractions were combined and used as final enzyme stock solution.

Production and purification of *Nc*LPMO9M have been described elsewhere.[6, 7]

Fractionation of Celluclast® 1.5 L and Accellerase® BG

Commercial enzyme cocktails of Celluclast® 1.5 L and Accellerase® BG were fractionated by using SEC in an ÄKTA-Micro chromatography system (GE Healthcare). Prior to the fractionation, Celluclast® 1.5 L and Accellerase® BG were washed three times with a 20 mM potassium phosphate buffer (pH 7.0) containing 150 mM NaCl using Amicon® ultra centrifugal filters (3 kDa cut-off, Sigma-Aldrich). Enzyme samples were loaded on a Superdex™ 75 Increase 10/300 GL column (100 × 3 mm diameter, GE Healthcare) and eluted (1 mL/min) with a 20 mM potassium phosphate buffer (pH 7.0) containing 150 mM NaCl. Peak fractions (based on UV 280 nm) of each enzyme cocktail were collected, combined and concentrated via ultrafiltration. The fractionated Celluclast® 1.5 L and Accellerase® BG were dialyzed with 50 mM sodium acetate buffer (pH 5.0) and subsequently stored at 4 °C.

Screening of Substrate Specificities of *Mt*9B$^+$ and *Mt*9B$^-$

All carbohydrate substrates and their tested combinations are listed in **Table 1**. Substrates (final concentration of 2 mg/mL each) were dissolved or suspended in 50 mM ammonium acetate buffer (pH 5.0) in the absence or presence of 1 mM ascorbic acid (Asc, final concentration). Subsequently, *Mt*9B$^+$ and *Mt*9B$^-$ were added to the corresponding mixtures (0.5 mL total volume) at a final concentration of 0.75 µM. Control samples either contained substrates and enzymes without Asc, or the different cellulose types with 1 mM Asc. The reaction was incubated in an Eppendorf Thermomixer® Comfort at 30 °C under shaking at 800 rpm, placed in an almost vertical direction, for 24 h. The incubation was stopped by separation of supernatant (SUP) from the residue (RES) directly after centrifugation at 22000

× g for 10 min at 4 °C. SUP was collected and stored at -20 °C. SUP was diluted five times prior to high performance anion exchange chromatography (HPAEC) analysis.

Binding of *Mt*9B$^+$ and *Mt*9B$^-$ to Different Cellulose Types

Binding experiments were performed according to Petrović et al.[8] and Courtade et al..[9] 5 μM *Mt*9B$^+$ or *Mt*9B$^-$ were added to 2 mg/mL BC, AVI or RAC in 50 mM ammonium acetate buffer pH 5.0. The protein-cellulose mixture was incubated at 30 or 50 °C in an Eppendorf ThermoMixer® Comfort (Eppendorf, Hamburg, Germany) at 800 rpm (in an almost vertical orientation) for 240 min. Subsequently, supernatant with free protein was separated and collected from residual cellulose with bound protein through centrifugation 22,000 ×g, 2 min at 4 °C in a table centrifuge. The amount of bound protein was calculated based on the following equation (eq.1):

Eq. 1 $$Bound\ protein\ (\mu M) = Total\ protein\ (\mu M) - Total\ protein\ (\mu M) \times \frac{Abs\ free\ (AU\ 280)}{Abs\ total\ (AU\ 280)}$$

where *Total protein* = 5 μM; *Abs free* = absorbance of free protein in the supernatant at 280 nm; *Abs total* = absorbance of total protein solution without substrate at 280 nm.

The absorbance of free protein and total protein was measured spectrophotometrically (SpectraMax M2e/iD3, Molecular Devices, USA) at 280 nm in a 96-well plate. Both the incubations and the measurements were performed in duplicate.

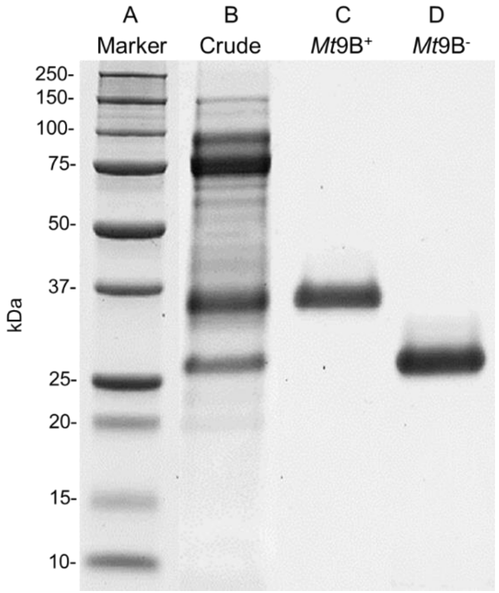

Figure S1. SDS-PAGE analysis of crude *Mt*9B$^+$ and *Mt*9B$^-$-containing fraction (lane B) and purified *Mt*9B$^+$ (lane C) and *Mt*9B$^-$ (lane D). The molecular masses (kDa) of marker proteins (A) are indicated.

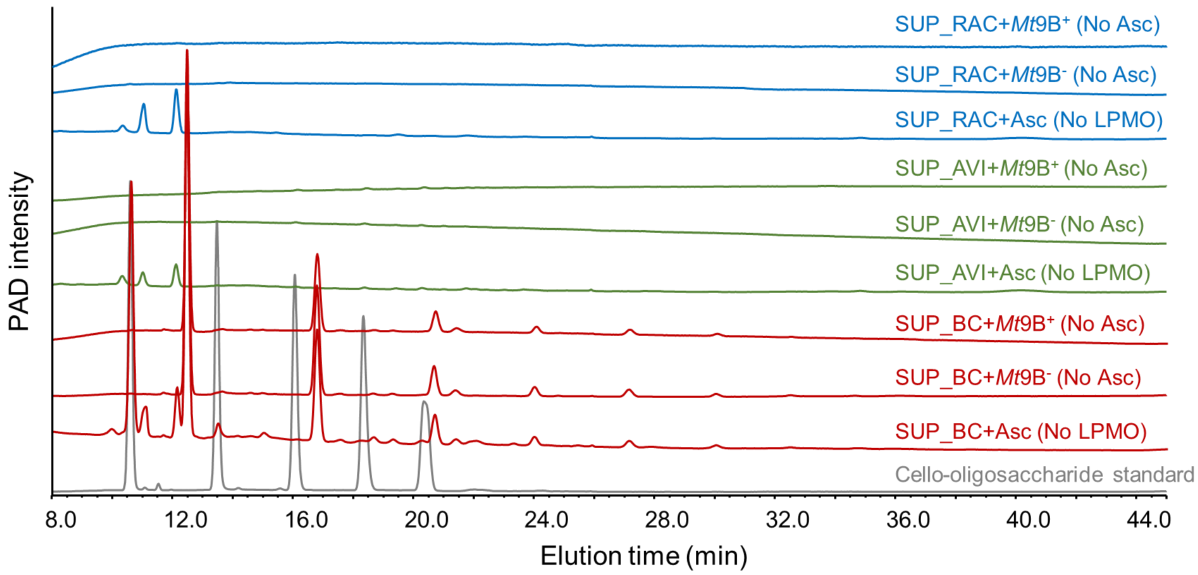

Figure S2. HPAEC elution patterns of control samples. In the absence of Asc, the supernatants from the incubation (24 h and 30 °C) of *Mt*9B$^+$ and *Mt*9B$^-$ with BC (SUP_BC+*Mt*9B$^+$ (no Asc) and SUP_BC+*Mt*9B$^-$ (no Asc)), AVI (SUP_AVI+*Mt*9B$^+$ (no Asc) and SUP_AVI+*Mt*9B$^-$ (no Asc)) and BC (SUP_RAC+*Mt*9B$^+$ (no Asc) and SUP_RAC+*Mt*9B$^-$ (no Asc)) were analyzed. The supernatants from the incubation of BC, AVI and RAC without enzymes but with Asc (SUP_BC+Asc (no LPMO), SUP_AVI+Asc (no LPMO) and (SUP_BC+Asc (no LPMO)) were also analyzed. A standard containing a mixture of cellobiose, cellotriose, cellotetraose, cellopentaose and cellohexaose (from left to right in chromatogram) is shown in gray. HPAEC elution patterns of control samples incubated at 30 °C and 50 °C were identical.

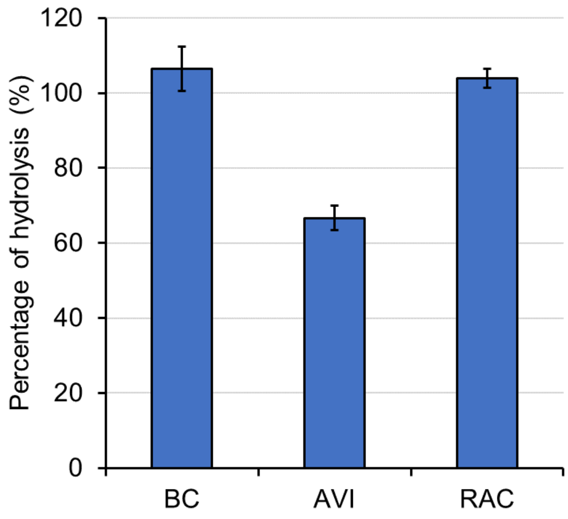

Figure S3. Percentage of hydrolysis of BC, AVI and RAC by using the fractionated cellulase cocktail of Celluclast® 1.5 L and Accellerase® BG. The percentage of hydrolysis was calculated based on the amount of glucose released (corrected by the weight difference between glucose and its anhydrous form) divided by the initial substrate weight. The hydrolysis of each substrate was performed in triplicate.

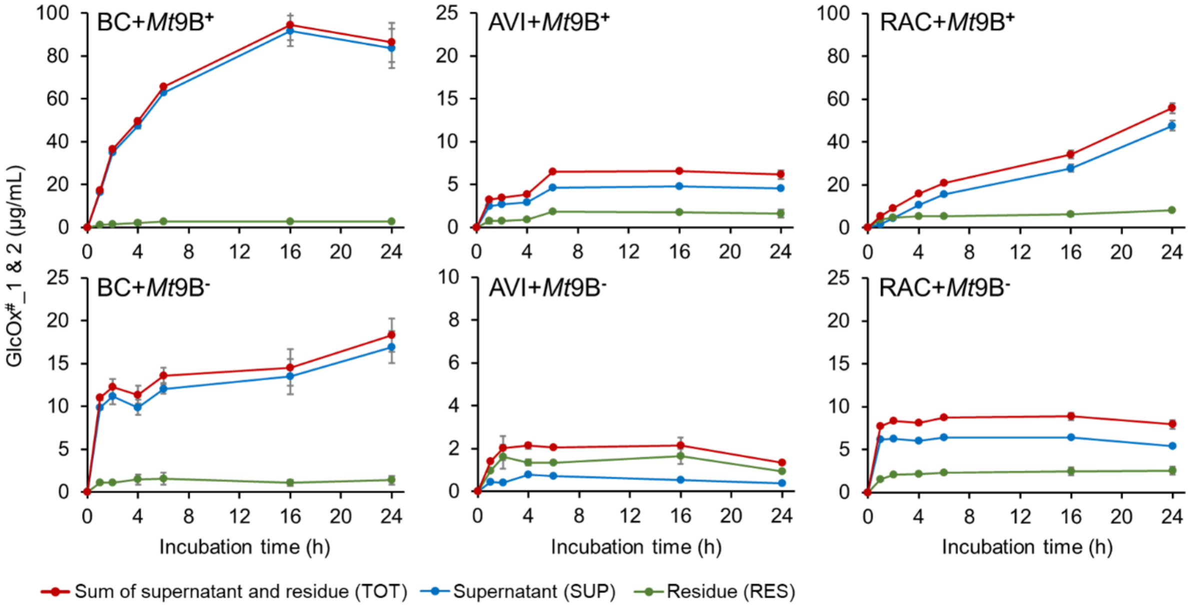

Figure S4. Amounts of gluconic acid (GlcOx#_1) and cellobionic acid (GlcOx#_2) generated from BC, AVI and RAC by *Mt*9B$^+$ and *Mt*9B$^-$ after subsequent hydrolysis, in supernatant (SUP, blue), residue (RES, green) and the sum of both (TOT, red) over time at 50 °C. Error bars (grey) indicate the standard deviations (± std) of duplicate measurements. Amount of GlcOx#_1 and GlcOx#_2 generated from BC, AVI and RAC by *Mt*9B$^+$ and *Mt*9B$^-$ after subsequent hydrolysis, in SUP, RES and TOT over time at 30 °C is shown in **Figure 1**.

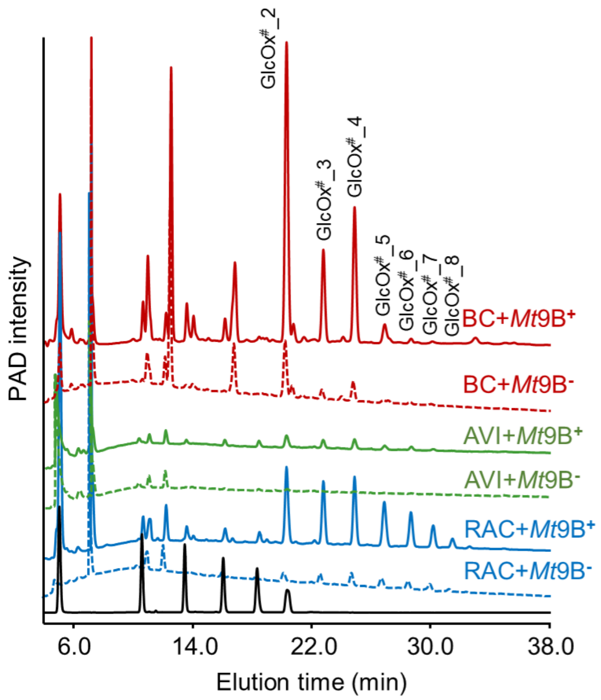

Figure S5. HPAEC elution patterns of supernatants (SUP) from BC, AVI and RAC digests (24 h incubation) of *Mt*9B$^+$ and *Mt*9B$^-$ in the presence of Asc at 50 °C. HPAEC elution patterns of supernatants generated at 30 °C are shown in **Figure 2**. Annotation of C1-oxidized cello-oligosaccharides (GlcOx#_2 to GlcOx#_8) is based on the previous study.[1, 5] A standard containing a mixture of glucose, cellobiose, cellotriose, cellotetraose, cellopentaose and cellohexaose (from left to right in chromatogram) is shown in black. SUP of control incubations is shown in **Figure S2**.

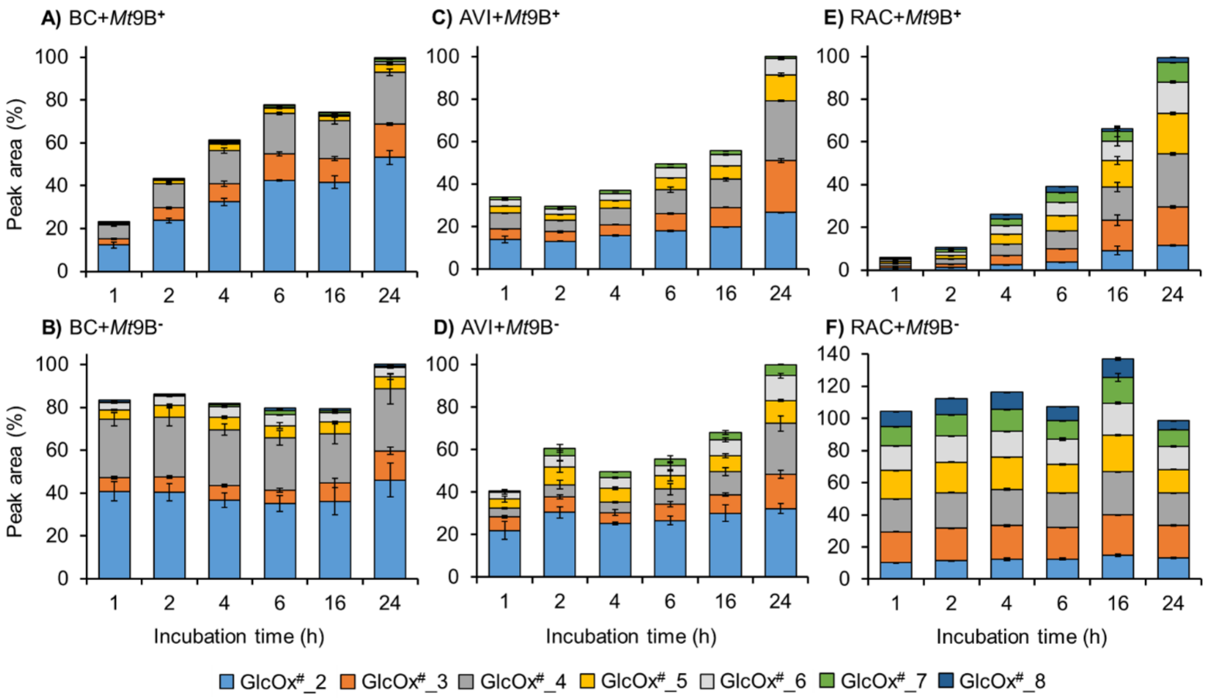

Figure S6. Relative quantification of peak area of each DP of oxidized cello-oligosaccharides (GlcOx$^{\#}$_2 to GlcOx$^{\#}$_8) generated by *Mt*9B^{+} and *Mt*9B^{-} from the time-course incubation with BC, AVI and RAC at 50 °C. Total peak area of each 24 h sample was set to 100%. Relative quantification of the peak area of released oxidized cello-oligosaccharides at 30 °C is shown in **Figure 3**.

Table S1. The presence of a CBM, regioselectivity and substrate specificity of four AA9 LPMOs used in this study. n.r. = not relevant

Name	CBM	Regioselectivity	Substrate specificity	Reference
*Mt*LPMO9B (*Mt*9B$^+$)	CBM1	C1	Cellulose	Frommhagen et al.[1] Grieco et al.[10]
*Mt*9B$^-$	No	Unknown	Unknown	n.r.
*Mt*LPMO9I	No	C1	Cellulose	Sun et al.[5]
*Mt*LPMO9H	CBM1	C1 and C4	Cellulose (only substrate tested)	Grieco et al.[10]
*Nc*LPMO9M	No	C1 and C4	Cellulose, xyloglucan	Phillips et al.[11] Sun et al.[12]

Table S2. Amounts of bound *Mt*9B$^+$ and *Mt*9B$^-$ on BC, AVI and RAC (total protein amount of 5 μM each)

Substrate	Bound (μM)			
	30 °C		50 °C	
	*Mt*9B$^+$	*Mt*9B$^-$	*Mt*9B$^+$	*Mt*9B$^-$
BC	1.08 ± 0.28	0.74 ± 0.19	1.42 ± 0.21	0.79 ± 0.07
AVI	1.34 ± 0.17	0.62 ± 0.21	1.08 ± 0.18	0.86 ± 0.22
RAC	2.85 ± 0.32	1.04 ± 0.11	2.90 ± 0.18	0.88 ± 0.21

Table S3. Amounts of gluconic acid and cellobionic acid (GlcOx#_1 & 2, µg/mL) generated from BC, AVI and RAC by *Mt*9B$^+$ and *Mt*9B$^-$ after subsequent hydrolysis, in supernatant (SUP), residue (RES) and the sum of both (TOT) over time at 30 °C. Error indicate the standard deviations (± std) of duplicate measurements

	BC+*Mt*9B$^+$						
	0 h	1 h	2 h	4 h	6 h	16 h	24 h
TOT	0 ± 0	9.6 ± 0.5	20.6 ± 1.5	32.4 ± 2.2	43.4 ± 3.0	70.8 ± 3.4	89.4 ± 7.9
SUP	0 ± 0	8.5 ± 0.4	19.6 ± 1.4	31.5 ± 2.2	41.8 ± 2.9	68.6 ± 3.4	87.5 ± 7.9
RES	0 ± 0	1.1 ± 0.3	1.0 ± 0.3	1.3 ± 0.3	1.6 ± 0.2	2.2 ± 0.4	2.1 ± 0.7
	BC+*Mt*9B$^-$						
	0 h	1 h	2 h	4 h	6 h	16 h	24 h
TOT	0 ± 0	12.7 ± 0.6	12.3 ± 1.3	13.7 ± 1.7	14.1 ± 1.3	16.2 ± 1.1	21.3 ± 0.0
SUP	0 ± 0	11.9 ± 0.5	12.0 ± 1.3	13.6 ± 1.7	13.5 ± 1.3	15.4 ± 1.1	20.7 ± 0.0
RES	0 ± 0	0.8 ± 0.2	0.3 ± 0.1	0.2 ± 0.1	0.7 ± 0.1	0.8 ± 0.1	0.6 ± 0.0
	AVI+*Mt*9B$^+$						
	0 h	1 h	2 h	4 h	6 h	16 h	24 h
TOT	0 ± 0	2.7 ± 0.2	4.4 ± 0.5	7.5 ± 0.3	9.7 ± 0.3	16.5 ± 0.0	19.5 ± 0.7
SUP	0 ± 0	1.7 ± 0.1	2.9 ± 0.1	5.9 ± 0.2	8.1 ± 0.2	14.7 ± 0.0	16.9 ± 0.0
RES	0 ± 0	1.0 ± 0.1	1.5 ± 0.5	1.6 ± 0.2	1.6 ± 0.3	1.8 ± 0.0	2.6 ± 0.7
	AVI+*Mt*9B$^-$						
	0 h	1 h	2 h	4 h	6 h	16 h	24 h
TOT	0 ± 0	1.6 ± 0.1	2.2 ± 0.2	2.9 ± 0.2	3.4 ± 0.2	3.4 ± 0.3	3.3 ± 0.3
SUP	0 ± 0	0.9 ± 0.1	1.5 ± 0.0	1.9 ± 0.1	2.0 ± 0.1	1.9 ± 0.1	2.1 ± 0.0
RES	0 ± 0	0.7 ± 0.0	0.7 ± 0.1	1.0 ± 0.1	1.4 ± 0.2	1.4 ± 0.2	1.2 ± 0.3
	RAC+*Mt*9B$^+$						
	0 h	1 h	2 h	4 h	6 h	16 h	24 h
TOT	0 ± 0	3.6 ± 0.3	4.1 ± 0.2	5.7 ± 0.4	6.0 ± 0.2	9.6 ± 0.5	14.3 ± 0.6
SUP	0 ± 0	1.7 ± 0.2	1.5 ± 0.2	2.3 ± 0.1	2.4 ± 0.1	5.2 ± 0.1	8.9 ± 0.5
RES	0 ± 0	1.9 ± 0.3	2.6 ± 0.1	3.4 ± 0.4	3.6 ± 0.2	4.4 ± 0.4	5.4 ± 0.3
	RAC+*Mt*9B$^-$						
	0 h	1 h	2 h	4 h	6 h	16 h	24 h
TOT	0 ± 0	5.3 ± 0.1	8.7 ± 0.4	13.9 ± 0.6	15.3 ± 0.4	13.9 ± 0.8	15.4 ± 0.4
SUP	0 ± 0	4.5 ± 0.1	7.4 ± 0.1	11.9 ± 0.1	13.2 ± 0.3	11.5 ± 0.8	13.0 ± 0.3
RES	0 ± 0	0.8 ± 0.0	1.3 ± 0.3	2.0 ± 0.6	2.2 ± 0.3	2.4 ± 0.2	2.4 ±0.2

Table S4. Amounts of gluconic acid and cellobionic acid (GlcOx[#]_1 & 2, μg/mL) generated from BC, AVI and RAC by *Mt*9B^{+} and *Mt*9B^{-} after subsequent hydrolysis, in supernatant (SUP), residue (RES) and the sum of both (TOT) over time at 50 °C. Error indicate the standard deviations (± std) of duplicate measurements

BC+*Mt*9B^{+}							
	0 h	1 h	2 h	4 h	6 h	16 h	24 h
TOT	0 ± 0	17.4 ± 0.2	36.6 ± 0.6	49.6 ± 1.6	65.6 ± 0.8	94.6 ± 7.2	86.4 ± 9.2
SUP	0 ± 0	16.3 ± 0.2	35.0 ± 0.5	47.5 ± 1.4	63.0 ± 0.8	91.7 ± 7.2	83.5 ± 9.2
RES	0 ± 0	1.1 ± 0.0	1.6 ± 0.3	2.1 ± 0.9	2.6 ± 0.0	2.9 ± 0.1	2.8 ± 0.1
BC+*Mt*9B^{-}							
	0 h	1 h	2 h	4 h	6 h	16 h	24 h
TOT	0 ± 0	11.0 ± 0.3	12.2 ± 0.9	11.3 ± 1.0	13.6 ± 0.9	14.5 ± 2.1	18.3 ± 1.9
SUP	0 ± 0	9.9 ± 0.2	11.1 ± 0.9	9.9 ± 0.9	12.1 ± 0.5	13.5 ± 2.1	16.9 ± 1.9
RES	0 ± 0	1.1 ± 0.2	1.1 ± 0.0	1.5 ± 0.6	1.5 ± 0.7	1.1 ± 0.4	1.4 ± 0.5
AVI+*Mt*9B^{+}							
	0 h	1 h	2 h	4 h	6 h	16 h	24 h
TOT	0 ± 0	3.2 ± 0.3	3.4 ± 0.1	3.8 ± 0.2	6.5 ± 0.1	6.6 ± 0.1	6.1 ± 0.5
SUP	0 ± 0	2.5 ± 0.0	2.7 ± 0.0	2.9 ± 0.1	4.6 ± 0.1	4.8 ± 0.0	4.5 ± 0.0
RES	0 ± 0	0.7 ± 0.3	0.8 ± 0.1	0.9 ± 0.1	1.9 ± 0.1	1.8 ± 0.1	1.6 ± 0.5
AVI+*Mt*9B^{-}							
	0 h	1 h	2 h	4 h	6 h	16 h	24 h
TOT	0 ± 0	1.4 ± 0.1	2.0 ± 0.6	2.1 ± 0.1	2.1 ± 0.1	2.2 ± 0.4	1.3 ± 0.1
SUP	0 ± 0	0.4 ± 0.0	0.4 ± 0.1	0.8 ± 0.0	0.7 ± 0.0	0.5 ± 0.0	0.4 ± 0.1
RES	0 ± 0	1.0 ± 0.1	1.6 ± 0.6	1.3 ± 0.1	1.3 ± 0.0	1.6 ± 0.4	0.9 ± 0.0
RAC+*Mt*9B^{+}							
	0 h	1 h	2 h	4 h	6 h	16 h	24 h
TOT	0 ± 0	5.5 ± 1.1	9.2 ± 0.8	15.9 ± 0.5	21.1 ± 0.7	34.2 ± 1.9	55.8 ± 2.3
SUP	0 ± 0	1.7 ± 1.1	4.4 ± 0.1	10.5 ± 0.3	15.6 ± 0.5	27.8 ± 1.7	47.7 ± 2.3
RES	0 ± 0	3.9 ± 0.4	4.8 ± 0.7	5.3 ± 0.4	5.4 ± 0.5	6.3 ± 1.0	8.1 ± 0.0
RAC+*Mt*9B^{-}							
	0 h	1 h	2 h	4 h	6 h	16 h	24 h
TOT	0 ± 0	7.7 ± 0.2	8.3 ± 0.2	8.1 ± 0.2	8.7 ± 0.1	8.9 ± 0.5	7.9 ± 0.5
SUP	0 ± 0	6.2 ± 0.1	6.3 ± 0.2	6.0 ± 0.1	6.4 ± 0.1	6.4 ± 0.1	5.4 ± 0.2
RES	0 ± 0	1.5 ± 0.2	2.0 ± 0.1	2.1 ± 0.1	2.3 ± 0.1	2.5 ± 0.4	2.5 ± 0.5

References

1. Frommhagen, M.; Koetsier, M. J.; Westphal, A. H.; Visser, J.; Hinz, S. W.; Vincken, J-P.; Berkel, W. J.; Kabel, M. A.; Gruppen, H., Lytic polysaccharide monooxygenases from *Myceliophthora thermophila* C1 differ in substrate preference and reducing agent specificity. *Biotechnol. Biofuels* **2016,** *9*, 186, DOI: 10.1186/s13068-016-0594-y

2. Frommhagen, M.; Sforza, S.; Westphal, A. H.; Visser, J.; Hinz, S. W.; Koetsier, M. J.; van Berkel, W. J.; Gruppen, H.; Kabel, M. A., Discovery of the combined oxidative cleavage of plant xylan and cellulose by a new fungal polysaccharide monooxygenase. *Biotechnol. Biofuels* **2015,** *8*, 101, DOI: 10.1186/s13068-015-0284-1

3. Punt, P. J.; Burlingame, R. P.; Pynnonen, C. M.; Olson, P. T.; Wery, J.; Visser, J.; Heinrich, J.; Emalfarb, M.; Visser, J.; Verdoes, J., *Chrysosporium lucknowense* protein production system. WO/2010/107303, 2010.

4. Visser, H.; Joosten, V.; Punt, P. J.; Gusakov, A. V.; Olson, P. T.; Joosten, R.; Bartels, J.; Visser, J.; Sinitsyn, A. P.; Emalfarb, M. A., Development of a mature fungal technology and production platform for industrial enzymes based on a *Myceliophthora thermophila* isolate, previously known as *Chrysosporium lucknowense* C1. *Ind. Biotechnol.* **2011,** *7*, 214-223, DOI: 10.1089/ind.2011.7.214

5. Sun, P.; Frommhagen, M.; Kleine Haar, M.; van Erven, G.; Bakx, E.; van Berkel, W.; Kabel, M., Mass spectrometric fragmentation patterns discriminate C1- and C4-oxidised cello-oligosaccharides from their non-oxidised and reduced forms. *Carbohydr. Polym.* **2020,** *234*, 115917, DOI: 10.1016/j.carbpol.2020.115917

6. Kittl, R.; Kracher, D.; Burgstaller, D.; Haltrich, D.; Ludwig, R., Production of four *Neurospora crassa* lytic polysaccharide monooxygenases in *Pichia pastoris* monitored by a fluorimetric assay. *Biotechnol. Biofuels* **2012,** *5*, 79, DOI: 10.1186/1754-6834-5-79

7. Laurent, C.; Sun, P.; Scheiblbrandner, S.; Csarman, F.; Cannazza, P.; Frommhagen, M.; van Berkel, W. J. H.; Oostenbrink, C.; Kabel, M. A.; Ludwig, R., Influence of lytic polysaccharide monooxygenase active site segments on activity and affinity. *Int. J. Mol. Sci.* **2019,** *20*, 6219, DOI: 10.3390/ijms20246219

8. Petrovic, D. M.; Bissaro, B.; Chylenski, P.; Skaugen, M.; Sorlie, M.; Jensen, M. S.; Aachmann, F. L.; Courtade, G.; Varnai, A.; Eijsink, V. G. H., Methylation of the N-terminal histidine protects a lytic polysaccharide monooxygenase from auto-oxidative inactivation. *Protein Sci.* **2018,** *27*, 1636-1650, DOI: 10.1002/pro.3451

9. Courtade, G.; Forsberg, Z.; Heggset, E. B.; Eijsink, V. G. H.; Aachmann, F. L., The carbohydrate-binding module and linker of a modular lytic polysaccharide monooxygenase promote localized cellulose oxidation. *J. Biol. Chem.* **2018,** *293*, 13006-13015, DOI: 10.1074/jbc.RA118.004269

10. Grieco, M. A. B.; Haon, M.; Grisel, S.; de Oliveira-Carvalho, A. L.; Magalhaes, A. V.; Zingali, R. B.; Pereira, N., Jr.; Berrin, J. G., Evaluation of the enzymatic arsenal secreted by *Myceliophthora thermophila* during growth on sugarcane bagasse with a focus on LPMOs. *Front. Bioeng. Biotechnol.* **2020,** *8*, 1028, DOI: 10.3389/fbioe.2020.01028

11. Phillips, C. M.; Beeson, W. T.; Cate, J. H.; Marletta, M. A., Cellobiose dehydrogenase and a copper-dependent polysaccharide monooxygenase potentiate cellulose degradation by *Neurospora crassa*. *ACS Chem. Biol.* **2011,** *6*, 1399-406, DOI: 10.1021/cb200351y

12. Sun, P.; Laurent, C.; Scheiblbrandner, S.; Frommhagen, M.; Kouzounis, D.; Sanders, M. G.; van Berkel, W. J. H.; Ludwig, R.; Kabel, M. A., Configuration of active site segments in lytic

polysaccharide monooxygenases steers oxidative xyloglucan degradation. *Biotechnol. Biofuels* **2020,** *13*, 95, DOI: 10.1186/s13068-020-01731-x
